# Association Between Comorbid Psychiatric Disorders and Persistent Smoking After a Diagnosis of Chronic Obstructive Pulmonary Disease Among Patients Seeking Treatment at a Tertiary Care Hospital in India

**DOI:** 10.7759/cureus.37688

**Published:** 2023-04-17

**Authors:** Mahendra S Uikey, Prabhoo Dayal

**Affiliations:** 1 Psychiatry, All India Institute of Medical Sciences, New Delhi, IND; 2 National Drug Dependence Treatment Centre, All India Institute of Medical Sciences, New Delhi, IND

**Keywords:** psychiatric comorbidities, copd: chronic obstructive pulmonary disease, comorbid anxiety, depression, smoking tobacco

## Abstract

Introduction

Smoking cessation is the most effective approach to slowing down the progression of chronic obstructive pulmonary disease (COPD). Despite this, almost half of COPD patients continue to smoke after diagnosis. COPD patients with current smoking status are more likely to have concurrent psychiatric comorbidities, for instance, depression and anxiety. These psychiatric disorders can contribute to the persistence of smoking in individuals with COPD. This study aimed to investigate predictors of smoking persistence in COPD patients.

Materials and methods

A cross-sectional study was conducted in the Outpatient Department (OPD) of the Department of Pulmonary Medicine in a tertiary care hospital from August 2018 to July 2019. Patients with COPD were screened for their smoking status. All subjects were then personally assessed for any psychiatric comorbidity using the Mini International Neuropsychiatric Interview (MINI), the Patient Health Questionnaire-9 (PHQ-9), and the Anxiety Inventory for Respiratory (AIR) Disease. Logistic regression was performed to compute the odds ratio (OR).

Results

The study included a total of 87 COPD patients. Of the 87 COPD patients, 50 were current smokers, and 37 were past smokers. COPD patients with psychiatric disorders were four times more likely to continue smoking than those without psychiatric comorbidities (OR: 4.62, 95% CI: 1.46-14.54). The results showed that increasing PHQ-9 scores by one unit in COPD patients increased the likelihood of continuing to smoke by 27 percent.

Conclusion

In our multivariate analysis, current depression was found as a significant predictor of continued smoking in COPD patients. The present results are consistent with reports from previous research that depressive symptoms are associated with continued smoking in patients with COPD. COPD patients who are currently smoking should be examined for psychiatric disorders and treated concurrently to achieve effective smoking cessation.

## Introduction

Tobacco use is known to be a leading cause of various illnesses and premature deaths across the world. It is well established that tobacco use causes cardiovascular and respiratory diseases. According to the World Health Organization (WHO), tobacco use is linked to over 20 different types or subtypes of cancer. Chronic obstructive pulmonary disease (COPD) is a chronic and progressive respiratory disease marked by incompletely reversible airflow obstruction known to cause respiratory symptoms like dyspnea, cough, and excessive sputum production [1]. According to the American Lung Association, smoking accounts for up to 90% of COPD cases, with smokers being 12 to 13 times more likely to die from COPD than nonsmokers. Additionally, the Global Initiative for chronic obstructive lung disease (GOLD) reports that smoking cessation is the most effective way to slow the progression of COPD and improve the quality of life for those with the disease. Despite the fact that quitting could have long-term benefits, however, some patients continued to smoke. Previous studies [2-4] have highlighted that sociodemographic and clinical predictor (such as nicotine dependence severity and gender) do not continue to predict long-term tobacco cessation outcomes. This suggests that factors other than these traditional predictors may be more important in predicting successful long-term tobacco cessation outcomes. Previous studies [5] have also shown that around 40%-45% of COPD patients continue to smoke tobacco despite having good motivation to quit. It suggests that the severity of dependence or lack of motivation to quit smoking may not be the only factor, but psychological factors such as anxiety sensitivity and anhedonia may also contribute [6,7].

It was reported in previous studies that COPD patients were more likely to have psychiatric comorbidities, which may contribute to smoking persistence [8,9]. Recent studies [10,11] have reported that distress intolerance, anxiety sensitivity, and anhedonia are important psychological traits that play a role in tobacco smoking, depression, and anxiety. These psychological factors can contribute to smoking persistence and exacerbate depression and anxiety symptoms, creating a vicious cycle that makes it difficult for even the most motivated people to quit.

This study aimed to examine the clinical and demographic features of current and past tobacco smokers seeking treatment for COPD and to identify the factors that predict smoking persistence in COPD patients. Therefore, identifying predictors of continued smoking can aid in the development of more effective interventions for COPD patients, including standard smoking cessation interventions combined with the management of anxiety and depression in those who are trying to quit smoking and who also have anxiety or/and depression comorbidities.

## Materials and methods

Type of study

A cross-sectional study was conducted in the Outpatients Department (OPD) of the Department of Pulmonary Medicine, All India Institute of Medical Sciences (AIIMS), New Delhi, from August 2018 to July 2019. A total of 87 patients aged 30 and older who were diagnosed with COPD were recruited through the purposive sampling approach.

Study subjects and eligibility 

Patients with COPD undergoing treatment at the Department of Pulmonary Medicine, All India Institute of Medical Sciences, New Delhi, India, were screened for smoking by being asked, “Have you smoked at least 100 cigarettes/bidis (or any other type) in your entire lifetime?” [12]. Patients meeting the inclusion criteria and showing their interest in participation were included after obtaining their informed consent. Inclusion criteria for the study were: male COPD patients aged 30 or above, who are current or past smokers, have been registered with the Pulmonary Department, AIIMS New Delhi, and have expressed a willingness to participate in the study. Exclusion criteria for the study were: patients with comorbid medical diseases (congestive heart failure, diabetes mellitus, and cerebrovascular accident), those who were taking oral corticosteroids, and patients who did not agree to participate. Female patients were excluded because the smoking rate among women in India is less than 2%.

Individuals who smoked at least one tobacco product daily in the last 30 days were considered current smokers [13]. Individuals with a history of daily smoking for at least a month or more and who did not smoke in the last 30 days were considered past smokers [13]. Ethical approval was received from the Institute Ethics Committee (Ref. No. IECPG-310/18.07.2018). Written informed consent was obtained from all study participants.

Measures

All subjects were further assessed in person by a psychiatry resident doctor using a semi-structured proforma, the mini international neuropsychiatric interview (MINI) [14], the tobacco use disorder module 7.0.2, the patient health questionnaire-9 (PHQ-9) [15], and the anxiety inventory for respiratory disease (AIR) [16]. The subjects with alcohol use were assessed using the alcohol use disorders identification test (AUDIT).

The Fagerstrom Test for Nicotine Dependence (FTND) and the Readiness to Change Questionnaire (RCQ) were used to assess current smokers for nicotine dependence and motivation to stop. Those who were found to have any psychiatric comorbidity were psychoeducated about the illness and treatment along with their accompanying family members, and then they were referred to the Department of Psychiatry. Those with a positive history of current alcohol and tobacco use were also psychoeducated about the illness and treatment, along with their family members, and subsequently referred to the Department of Psychiatry. In the COPD outpatient clinic, each patient had a proper medical record, including socio-demographic characteristics, medical history, results of a physical examination, lab test results, a pulmonary function test (PFT), and COPD assessment records. The severity of COPD was determined using the Global Initiative for Chronic Obstructive Lung Disease (GOLD) staging system, which is based on the results of pulmonary function tests (PFTs) and symptoms. The GOLD staging system classifies COPD into four stages.

Statistical analysis

The quantitative variables were summarized by the descriptive statistics mean (SD) for the normally distributed data and median (Inter Quartile Range) for the non-normally distributed data. The Kolmogorov-Smirnov (KS) test was used to test for the normality of the continuous variables in your study. The summarization of categorical variables was done using frequency and percentages. The association of categorical variables between two groups (current smokers and past smokers) was tested using the chi-square test/Fisher‘s exact test. The association of quantitative variables between two groups (current smokers and past smokers) was assessed by an independent t-test. The Mann-Whitney U test was used to report the association between variables that were not normally distributed. The two-sided p-value of less than 0.05 was considered to be statistically significant.

We used binary logistic regression to analyze the association between psychiatric disorders and smoking persistence in patients with COPD. The odds ratio (OR) was calculated after adjustment for covariates selected (p<0.05) from univariate models, including age, educational level, residential area, other medical comorbidities, and type of smoking. The analysis of the data was carried out by IBM Corp. Released 2011. IBM SPSS Statistics for Windows, Version 20.0. Armonk, NY: IBM Corp.

## Results

The study included a total of 87 COPD patients. Out of 87 patients with COPD, 50 were current smokers, and 37 were past smokers. The mean age of the patients was 58.87 ± 9.82 years, with no statistically significant age difference between current and past smokers (60.10 ± 10.73 vs. 57 ± 8.24, p= 0.14). There was no difference in the severity of COPD between current and past smokers. The current smokers were either more illiterate than past smokers (22% vs. 10.8%) or possessed a higher level of education (62%) than past smokers (44.9%). The difference in educational level was statistically significant (χ2=8.25, p=.016). Current smokers with COPD were more urban habituating than past smokers (70% vs. 51.4%, p =.076). However, there was no significant difference in a residential area between current smokers and past smokers who smoked more cigarettes (32% vs. 8.1%, p =.020) than past smokers with COPD. Current smokers had a higher proportion of medical co-morbidities than former smokers (52% vs. 27%, p = .016). Current alcohol use (26% vs. 8.1%, p = .03) and current psychiatric disorders (50% vs. 24.3%, p= .015) were also higher in current smokers with COPD than in past smokers. Current smokers were more likely to have a current depressive disorder than past smokers (38% vs. 13.5%, p=.012). However, there was no significant difference in lifetime depression between current smokers and past smokers. Current smokers with COPD were statistically not significant but showed a trend towards any anxiety disorder than in past smokers with COPD (24% vs. 8.1%, p =.052). Current smokers had a higher mean PHQ-9 score (5.90 ± 5.92 vs. 2.46 ±2.82, p=0.018) than past smokers with COPD (Table 1). 

**Table 1:** General features of the study sample by smoking status ^A^calculated by using the Pearson chi-square test/.@ Fisher’s Exact Test for categorical variables and Mann-Whitney U Test for continuous variables. ^C^PHQ-9: Patient Health Questionnaire-9; ^D^COPD: Chronic Obstructive Pulmonary Disease; ^B^GOLD: Global Initiative for Chronic Obstructive Lung Disease Classification

Variables	Total (n=87)	Current smokers (n=50)	Past smokers (n=37)	P^A^
	Mean (SD) or n(%)	Mean (SD) or n (%)	Mean (SD) or n (%)	
Age	58.78(9.82)	60.10(10.73)	57(8.24)	.147
Education Level				.016
No formal education	15(17.2)	11(22.0)	04(10.8)	
Up to secondary	24(27.6)	08(16.0)	16(43.2)	
Higher than secondary	48(55.2)	31(62.0)	17(45.9)	
Occupation (skill levels)				.891
Low	25(28.7)	14(28.0)	11(27.9)	
Medium	31(35.6	19(38.0)	12(32.4)	
High	31(35.6)	17(34.0)	14(37.8)	
Residential Area				.076
Urban	54(62.1)	35(70.0)	19(51.4)	
Rural	33(37.9	15(30.0)	18(48.6)	
^D^COPD Status				. 641
^B^GOLD 1	23(26.4)	12(24.0)	11(27.9)	
GOLD 2	42(48.3)	24(48.0)	18(48.6)	
GOLD 3	19(21.8)	13(26.0)	06(16.2)	
GOLD 4	03(3.4)	01(02.0)	02(05.4)	
Other Medical Co-morbidities				.019
Present	36(41.37)	26(52.0)	10(27.0)	
Absent	51(58.62)	24(48.0)	27(73.0)	
Type of Smoking				.020
Cigarettes	19(21.8)	16(32.0)	03(08.1)	
Beedi	68(78.2)	34(68.0)	34(91.9)	
Abstinence attempts	1.83(1.55)	1.92(1.72)	1.70(1.30)	.79
Any Anxiety Disorder				.052
Absent	72(82.8)	38(76.0)	34(91.9)	
Present	15(17.2)	12(24.0)	03(08.1)	
^C^PHQ –9 Score	4.44 (5.12)	5.90(5.92)	2.46(2.82)	.018
Lifetime Depressive Disorder				.112
Present	30(34.5)	21(42.0)	09(24.3)	
Absent	57(65.5)	29(58.0)	28(75.7)	
Current Depressive Disorder				.012
Present	24(27.6)	19(38.0)	05(13.5)	
Absent	63(72.4)	31(62.0)	32(86.5)	
Current Psychiatric Disorder Diagnosis				.015
Present	34(39.1)	25(50.0)	9(24.3)	
Absent	53(60.9)	25(50.0)	28(75.7)	

The result of the binary logistic regression was summarized in Table 2, taking into account the dependent variables current smokers and past smokers. In multivariable logistic regression, current depression, current psychiatric disorder, and any anxiety disorder were adjusted for age, level of education, place of residence, type of tobacco smoking product, and other medical comorbidities. The diagnosis of a current psychiatric disorder was significantly associated with continued smoking after adjustment for age, educational level, residential area, other medical comorbidities, and type of smoking product (OR: 4.62, 95% CI: 1.46-14.50). The current depression diagnosis was significantly associated with continued smoking after adjustment for age, educational level, residential area, other medical comorbidities, and type of smoking product (OR: 4.50, 95% CI: 1.34-15.06). Diagnoses of lifetime depressive disorder and any anxiety disorders were not associated with continued smoking after adjustment (Table 2). The Sidak correction was used to adjust the significance level after Sidak correction for the multiple corrections {((1-(1-0.05))^1/3)=0.016, three independent predictors in multivariable analysis}, only two variables remained significant. 

**Table 2:** Association of psychiatric disorders and odds of continued (persistent) smoking in patients with COPD AOR = adjusted odds ratio; COPD = chronic obstructive pulmonary disease; aOR = unadjusted odds ratio. bAdjusted for covariables selected (p<0.20) from univariate models, including age, educational level, residential area, other medical comorbidities, and type of smoking. The reference group for each category is the absence of the specified psychiatric disorder.

Psychiatric disorders	^a^OR(95%CI)	p-value	^b^AOR (95% CI)	p-value
Lifetime depression	2.52 (0.88-5.7)	0.091	2.79 (0.96-8.06)	0.058
Current depression	3.92 (1.30-11.80)	0.012	4.50 (1.34-15.06)	0.010
Any anxiety disorder	3.57 (0.93-13.76)	0.061	3.47 (0.80-15.02)	0.091
Current psychiatric disorder	3.11 (1.22-7.91)	0.012	4.62 (1.46-14.54)	<0.001

## Discussion

Chronic obstructive pulmonary disease (COPD) is a serious public health issue across the world. Tobacco smoking is the leading cause of COPD, so this observational study examined the predictors of continued smoking in COPD patients. In this study, COPD patients were grouped according to their current smoking status. The mean age of the total sample was 59.97 ± 10.12 years, which is comparable to most studies on COPD, which range from 54-67 years of age [17]. Current smokers with COPD and past smokers with COPD did not differ significantly in their level of education in the current study. Also, the product type of smoking did not remain statistically significant in multivariable regression. Previous studies found that smoking bidi was more likely to cause chest symptoms and lung impairment than smoking cigarettes [18]. Therefore, tobacco smokers (such as bidi smokers [bidi is a type of cigarette that is commonly used in South Asia, particularly in India and Bangladesh. It is made by rolling tobacco in a tendu or temburni leaf, which is a type of leaf commonly used in Indian cuisine]), who have a low perception of illness severity, negative consequences, and doubts about the benefits of quitting can benefit from interventions that focus on awareness, perceptions of the severity, potential exposure to the negative effects of bidi smoking, and the potential benefits of quitting.

In our multivariable analysis, comorbid current psychiatric disorders were a significant predictor of smoking persistence. Our results showed that current smokers were more likely to have psychiatric disorders than past smokers (OR: 4.62, 95% CI: 1.46-14.50). However, the current study found no significant association with other co-morbid medical illnesses in current smokers compared to past smokers. The results of the current study on psychiatric co-morbidity were comparable to previous studies [8,19,20]. In the current study, lifetime depression and any anxiety disorder were found to be 34.5% and 21.8%, respectively. These results were found to be consistent with previous studies, which reported that patients with COPD were more likely to experience depression and anxiety disorders than the general population [9,21,22]. This can be partly explained by the fact that both smoking and COPD increase the risk of depression and anxiety symptoms.

However, our results did not support findings from previous research that found an association between anxiety symptoms and an impaired ability to quit smoking [23]. In a meta-analysis, Jiang et al. [24] found that anxiety disorders had significant associations with smoking. In univariate analysis, lifetime depressive disorder was not statistically significant, which included current and past histories of depression. However, current depression was statistically significant in the univariate analysis and also remained a significant independent predictor of smoking persistence in the multivariable logistic regression (Table 2). The results of our study are in line with previous research that showed that individuals with current depression were more likely to have lower cessation rates, higher rates of relapse, and continued smoking than people without current depressive symptoms [10,25]. In a meta-analysis, Hitsman et al. [26] concluded that there was no association between depression history and tobacco cessation outcome. The continued smokers with COPD in our study were highly motivated and had a comparable number of attempts to quit smoking as past smokers. It suggests that current depressive symptoms may interfere with the smoking cessation process, which can lead to continued smoking in patients with COPD. In a systematic review, Mathew et al. [27] suggested that internal mood states such as increased negative affect and low positive affect can increase the positive outcome expectations for smoking in depressed smokers, which can further be increased during abstinence attempts. According to recent systematic reviews, distress intolerance, anxiety sensitivity, and anhedonia are important psychological traits that play a role in tobacco smoking, depression, and anxiety. These psychological factors can contribute to smoking persistence and exacerbate depression and anxiety symptoms [7,10,11], creating a vicious cycle that makes it difficult for even the most motivated people to quit. Consistent with previous research findings, our results also revealed that there was no difference in abstinence attempts between current smokers and past smokers in our study. The continued smokers with COPD in our study were highly motivated and had a comparable number of attempts to quit smoking as the past smokers. It suggests that current depressive symptoms may have an impact on successful smoking cessation through various psychological factors such as low positive affect or high negative affect and anhedonia in patients with COPD. Also, patients with current depressive symptoms may continue smoking to regulate these internal mood states. This could be one of the possible explanations for the persistence of smoking in depressed COPD patients compared to COPD patients without current depression. In a Cochrane review and meta-analysis by van der Meer et al. [28], smokers with current depression showed an improvement in tobacco cessation when standard smoking cessation interventions were combined with management of adverse mood states. Therefore, it is recommended that COPD patients who are currently smoking be examined for psychiatric disorders and treated concurrently to achieve effective smoking cessation. In this study, alcohol use and alcohol use disorder were found to be 18.3% and 8%, respectively, in COPD patients. The results of the current study on alcohol use and alcohol use disorder are consistent with a nationwide survey of drug use prevalence in India. The literature shows that patients with COPD who use alcohol while smoking have unsuccessful attempts to quit and low motivation to quit [29].

Strengths and limitations

A few limitations must be taken into account when interpreting the results of the present study. For example, only patients seeking treatment for COPD were included in the sample by using a purposive sampling method. Therefore, the result of the study cannot be generalized to all COPD patients. In this study, the Mini-International Neuropsychiatric Interview (MINI) was used for the ICD-10 psychiatric disorder assessment and is a more valid diagnostic tool for psychopathology assessment.

## Conclusions

Current depression disorder was found to be a significant predictor of continued smoking in patients with COPD in our multivariable analysis. The present results are consistent with reports from prior research that current depressive symptoms are associated with continued smoking. The continued smokers with COPD in our study were highly motivated and had a comparable number of attempts to quit smoking as the past smokers. It suggests that current depressive symptoms may have an impact on successful smoking cessation through various psychological factors such as low positive affect or high negative affect and anhedonia in patients with COPD. Therefore, it is recommended that COPD patients who are currently smoking be evaluated for psychiatric issues and treated concurrently to achieve successful smoking cessation. The majority of smoking cessation literature originates from developed countries, where cigarettes are the most common tobacco product. Tobacco cessation strategies in South Asian countries may need to be tailored to the tobacco products used there. The future study should be a follow-up study that observes how smoking changes over time and how psychiatric comorbidity affects smoking.
